# Dynamic Transcriptome Analysis of Anther Response to Heat Stress during Anthesis in Thermotolerant Rice (*Oryza sativa* L.)

**DOI:** 10.3390/ijms21031155

**Published:** 2020-02-10

**Authors:** Gang Liu, Zhongping Zha, Haiya Cai, Dandan Qin, Haitao Jia, Changyan Liu, Dongfeng Qiu, Zaijun Zhang, Zhenghuang Wan, Yuanyuan Yang, Bingliang Wan, Aiqing You, Chunhai Jiao

**Affiliations:** Hubei Key Laboratory of Food Crop Germplasm and Genetic Improvement, Food Crops Institute, Hubei Academy of Agricultural Sciences, Wuhan 430064, China; liug1112@163.com (G.L.); zhongpingzha@163.com (Z.Z.); ytchy@126.com (H.C.); hnqdd@163.com (D.Q.); jiahaitao.1986@163.com (H.J.); Liucy0602@163.com (C.L.); qdflcp@163.com (D.Q.); zjzhang0459@aliyun.com (Z.Z.); zhwan168@163.com (Z.W.); YYY681205@126.com (Y.Y.); ricewanbl@126.com (B.W.); aq_you@163.com (A.Y.)

**Keywords:** rice, heat stress, transcriptome, anther, anthesis

## Abstract

High temperature at anthesis is one of the most serious stress factors for rice (*Oryza sativa* L.) production, causing irreversible yield losses and reduces grain quality. Illustration of thermotolerance mechanism is of great importance to accelerate rice breeding aimed at thermotolerance improvement. Here, we identified a new thermotolerant germplasm, SDWG005. Microscopical analysis found that stable anther structure of SDWG005 under stress may contribute to its thermotolerance. Dynamic transcriptomic analysis totally identified 3559 differentially expressed genes (DEGs) in SDWG005 anthers at anthesis under heat treatments, including 477, 869, 2335, and 2210 for 1, 2, 6, and 12 h, respectively; however, only 131 were regulated across all four-time-points. The DEGs were divided into nine clusters according to their expressions in these heat treatments. Further analysis indicated that some main gene categories involved in heat-response of SDWG005 anthers, such as transcription factors, nucleic acid and protein metabolisms related genes, etc. Comparison with previous studies indicates that a core gene-set may exist for thermotolerance mechanism. Expression and polymorphic analysis of agmatine-coumarin-acyltransferase gene *OsACT* in different accessions suggested that it may involve in SDWG005 thermotolerance. This study improves our understanding of thermotolerance mechanisms in rice anthers during anthesis, and also lays foundation for breeding thermotolerant varieties via molecular breeding.

## 1. Introduction

Rice (*Oryza sativa* L.) is one of the most important and widely cultivated crops for global food security. However, rice farming is constantly subjected to various abiotic and biotic stresses; heat stress is a major abiotic stress that significantly affects rice growth and development [1,2]. It is estimated that rice grain yields decline by 10% for each 1 °C increase in minimum temperature during the growing season [3]. A report from the Intergovernmental Panel on Climatic Change (IPCC) predicted that, by the end of this century, average surface temperatures would increase by approximately 2.0–4.5 °C [4] (p. 151). Therefore, there is an urgent need to develop rice varieties with thermotolerance to cope with global climate change.

Rice production is particularly susceptible to high temperature, especially during the flowering and grain-filling stages, which directly affects grain yields and quality [2,5]. Even a short period of high temperature during these stages, such as >35 °C for 5 d at anthesis, could cause sterility [6,7]. Anther dehiscence is one of the most sensitive physiological processes affected by high temperature during anthesis; an increase in the basal pore length in a dehisced anther is critical for successful pollination [8,9]. Furthermore, differences in pollen numbers and germinating pollen and spikelet fertility between different rice genotypes have been associated with different levels of thermotolerance [8,10], leading to differences in yield under high-temperature stress.

Therefore, a comprehensive understanding of the mechanisms of thermotolerance at the reproductive stage is crucial for developing heat-tolerant varieties that are adapted to global warming. Many physiological studies have contributed to the understanding of heat responses during anthesis—one of the most heat sensitive stage—but molecular data are lacking.

Transcriptomics have been used to study the molecular mechanisms of thermotolerance in wheat [11], tomato [12], potato [13], and carnation [14]; consequently, multiple genes and pathways have been identified as heat-responsive. This information has helped us to understand how plants sense and respond to heat stress. In rice, some transcriptomic analyses have been conducted to investigate heat responses at the flowering stage [1,15,16,17,18,19]. However, most of these analyses were performed in tissues, such as spikelets or flag leaves, with only a few conducted on anthers or pistils [16,17]. To further clarify heat responses at the molecular level, additional studies are needed.

SDWG005 is a landrace from Africa that was identified as thermotolerant in previous study by our group [20]; its relative seed setting rate in the heat treatment (38 °C) was 98.5% of that in the control (28 °C). SDWG005 performs much better than N22, a well-known heat-tolerant rice germplasm, which relative seed setting rate was 64%–86% at 38 °C [5]. Rice variety 9311 is heat-sensitive based on its relatively lower seed setting rate (31.2%). By observing the morphology and microstructure of anthers in SDWG005 and 9311 before and after heat stress at anthesis, the anthers of SDWG005 were more tolerant to heat stress than those of 9311. To illustrate the key molecular mechanism underlying the thermotolerance of SDWG005, we conducted a transcriptional profile analysis under different time courses of heat treatment on the anthers of SDWG005 at anthesis based on RNA-seq. The findings reported here not only provide additional information for understanding the mechanisms of thermotolerance in rice at the reproductive stage but also lay the foundation for breeding heat-tolerant rice varieties. By using this germplasm, rice varieties with better thermotolerance could be developed through modern molecular breeding strategies.

## 2. Results

### 2.1. Thermotolerance Assay of SDWG005 and 9311 in Growth Chamber

**Figure 1:**
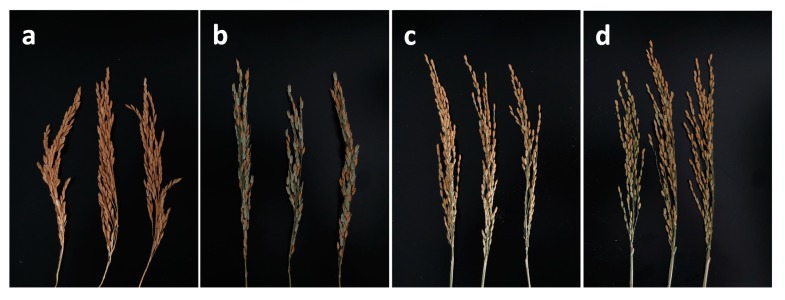
Mature spikes of 9311 and SDWG005 in the control (28 °C for 12 h per day) and heat treatment (38 °C for 12 h per day, for 5 days) conditions. (**a**,**b**) Panicles of 9311 plants under normal and heat stress conditions, (**c**,**d**) Panicles of SDWG005 plants under control and heat stress conditions.

Our previous field work has identified SDWG005 as thermotolerant but 9311 as thermosensitive. In this study, a growth chamber was used to mimic high temperature treatment on SDWG005 and 9311. The results showed that 9311 had more than 60% sterile spikelets after 5 d of heat treatment (relative seed setting rate 43.8%). In contrast, the spikelets of SDWG005 did not significantly differ between the control and heat treatment (relative seed setting rate 96.4%) (Figure 1). These findings suggested that SDWG005 was heat tolerant and 9311 was heat susceptible. 

### 2.2. Microstructure of Anthers in Thermotolerant SDWG005 under Heat Stress

**Figure 2:**
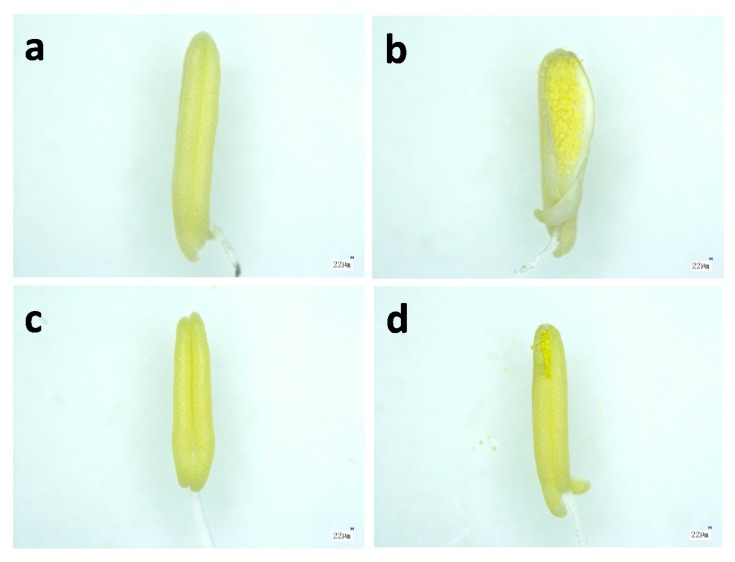
Anther morphology of SDWG005 and 9311 in the control and heat (38 °C for 12 h) treatments. Anthers of 9311 plants in the (**a**) control and (**b**) heat treatments, and anthers of SDWG005 plants in the (**c**) control and (**d**) heat treatments. Scale bar = 22 µm.

To examine the status of rice anthers under heat stress, morphology and microstructure of SDWG005 and 9311 anthers under normal conditions and after 12 h of heat treatment were observed under a microscope (Figure 2). As shown in Figure 2, there were no open anthers in either genotype under normal conditions (Figure 2a,c). After 12 h of heat treatment, the anthers of SDWG005 opened slightly (Figure 2d). In contrast to SDWG005, 9311, as a heat-sensitive variety at anthesis, showed widely opened anthers after the same treatment, but there was no pollen released from these opened anthers (Figure 2b).

**Figure 3:**
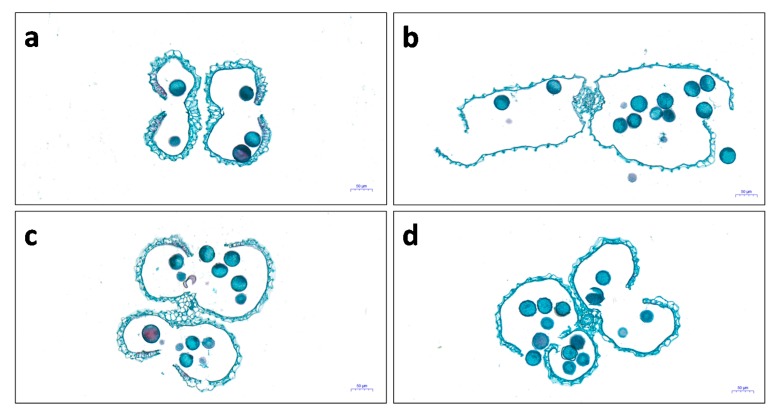
Microstructure of anthers of SDWG005 and 9311 in the control and after 12 h of heat treatment. Anthers of 9311 plants in the (**a**) control and (**b**) heat treatments, and anthers of SDWG005 plants in the (**c**) control and (**d**) heat treatments. Scale bar = 50 μm.

In addition, the walls of the anther sacs of 9311 had wilted significantly after 12 h of heat treatment, which damaged the anther structure due to water loss (Figure 3a,b). In contrast, the anthers of the tolerant genotype SDWG005 remained intact under heat stress (Figure 3c,d) and did not differ from those in the control.

### 2.3. Quality of RNA-Seq Data

To investigate the transcriptional profiles of heat stressed and unstressed anthers of SDWG005, 15 RNA samples representing four different time points (1, 2, 6, and 12 h) under heat treatment (38 °C) and one control (28 °C) were sequenced individually with Illumina technology; the generated reads were used to assemble the transcriptome for each sample. Approximately 7.17 Gb of data (at least 20 million clean reads, Q30 > 93.2%) were obtained for each sample. The clean reads were used to assemble the transcriptome for each sample by mapping the reads to rice reference genome (MSU7.0). More than 90.0% of the reads could be mapped to the rice reference genome, and approximately 92% and 2% of the mapped reads were mapped to exon and intron regions, respectively. The mapped reads from all samples were then remapped to the reference genome; 1629 new genes/transcripts were discovered.

### 2.4. Identification and Validation of DEGs

To adapt to stresses such as drought and high temperature, plants usually actively regulate the expression of endogenous genes. To investigate the dynamic transcriptional profiles in response to heat in anthers of SDWG005, changes in the expression of gene transcripts during heat treatment (including WD_1, WD_2, WD_6, WD_12, and WD_0 as a control) were profiled at the genome-wide level by RNA-seq. The expression level of each transcript/gene under each treatment (e.g., WD_1) was identified by the mean value of three biological replicates (e.g., WD_1_1, WD_1_2 and WD_1_3). All of the correlation coefficients between the three biological replicates for each treatment based on all of the transcripts were more than 0.9, indicating that the expression data were highly reproducible (Table S1).

**Figure 4:**
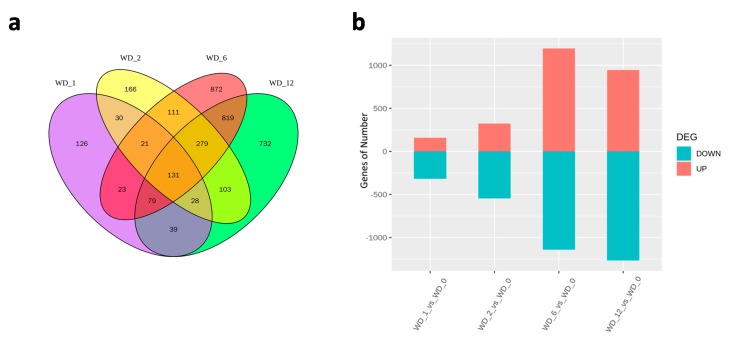
Analysis of differentially expressed genes in response to heat stress. (**a**) Venn diagram analysis of significantly differentially expressed genes at the four heat stress time points. (**b**) Numbers of significantly regulated genes at the four heat stress time points. Red and green columns represent the number of up- and down-regulated genes at different heat stress time points, respectively.

Genes that showed at least a two-fold change in expression in the heat-treated samples compared to the control sample were referred to as DEGs in this study. In total, 3559 genes were identified as DEGs at a minimum of one time-point. Based on the 3559 DEGs, three biological replicates for each sample could be clustered together by hierarchical cluster analysis (Figure S1), which indicated that the DEGs identified were reliable. The number of upregulated and downregulated DEGs varied considerably in different treatments. An increase in the number of DEGs was clearly observed as the heat treatment period lengthened, with 477, 869, 2335, and 2210 DEGs identified after 1 (WD_1), 2 (WD_2), 6 (WD_6), and 12 (WD_12) h of heat treatment, respectively (Figure 4a). All four heat treatments produced more downregulated genes than upregulated genes, especially in the short-term heat treatments (Figure 4b).

**Figure 5:**
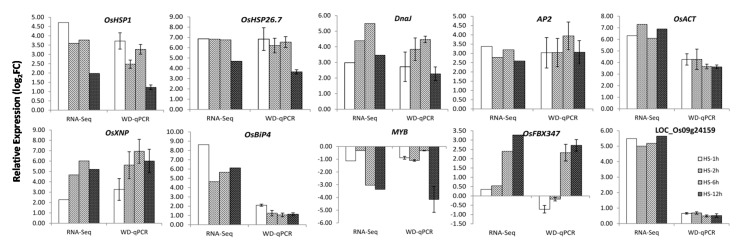
Expression levels of ten randomly selected DEGs by RNA-seq and qRT-PCR. RNA-seq: expression change of these genes in anthers of SDWG005 under heat treatments as compared to control by RNA-seq. WD-qPCR: expression change of these genes in anthers of SDWG005 under heat treatments as compared to control by real-time PCR.

To further validate the RNA-seq results of heat stress responsive genes in this study, qRT-PCR was conducted on anthers of SDWG005 under the same heat treatments as for RNA-seq to examine expressions of ten randomly selected DEGs. The expression patterns of all ten genes examined by qRT-PCR coincided with those determined by RNA-seq analysis (Figure 5), also indicating that the RNA-seq analysis was reliable. For example, RNA-seq analysis revealed 2–4.5 times higher expression levels of LOC_Os04g01740 (*OsHSP1*) in the heat shock (HS) samples than in the control, and the qRT-PCR analysis revealed 1.5–4 times higher expression levels of this gene relative to the control.

### 2.5. DEGs in Response to Different Heat Treatment Exposure

The 3559 DEGs were further compared in all four heat treatments; 210, 542, and 1308 common DEGs were found between WD_1 and WD_2, WD_2 and WD_6, and WD_6 and WD_12, respectively (Figure 4a). 

**Figure 6:**
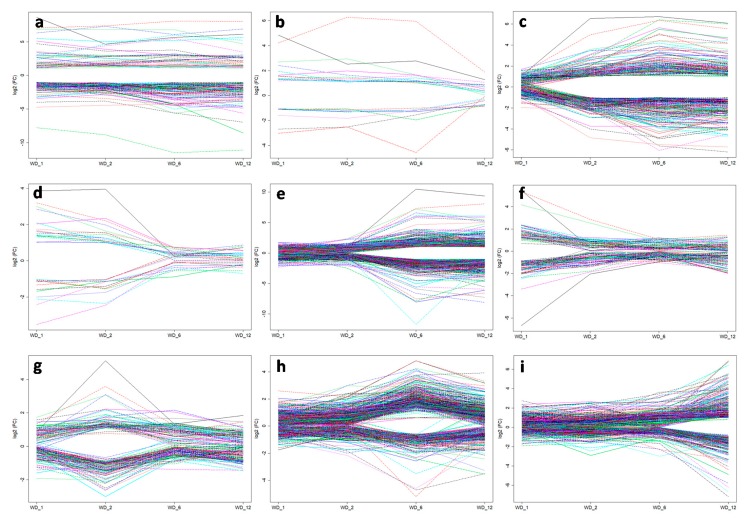
Expression of genes in different clusters. (**a**) cluster 1, genes regulated by all treatments; (**b**) cluster 2, genes regulated by short and intermediate heat treatments but not by long-term treatments; (**c**) cluster 3, genes not regulated by 1 hour heat shock, but by longer time of heat treatments after that; (**d**) cluster 4, genes regulated by the two short-term heat treatments, but not by the intermediate and long-term heat treatment; (**e**) cluster 5, genes regulated by intermediate and long-term heat treatments, but not by short-term heat treatments; (**f–i**) clusters 6–9, genes specifically regulated by 1, 2, 6, and 12 h of heat treatment, respectively. Different color lines indicated different genes.

To further investigate the dynamic expression of the 3559 DEGs at different time points of heat treatment, heatmap was constructed using *R* package firstly (Figure S2), and then genes with similar expression pattern were plotted using *R* language (Figure 6). As shown in Figure 6, the 3559 DEGs were mainly classified into nine clusters according to their expression level changes in the four heat treatments as compared to the control. Cluster 1 contained 129 DEGs that were shared across all treatments, accounting for less than 4% of the total DEGs. Further analysis showed that 33 and 96 of these genes were consistently up- or down-regulated, respectively, with different fold changes from 1 to 12 h of heat treatment. Cluster 2 contained 20 genes that were regulated by short and intermediate heat treatments but not by long-term treatments, while Cluster 3 contained 279 genes that were not regulated by 1 h heat shock (WD_1), but by longer time of heat treatments after that (WD_2, WD_6, and WD_12). Cluster 4 contained 30 genes that were regulated by the two short-term heat treatments, but not by the intermediate and long-term heat treatment. Cluster 5 contained 819 genes that were regulated by the intermediate and long-term heat treatments, but not by the short-term heat treatments. Clusters 6 to 9 contained 126, 166, 872, and 732 DEGs were identified to be specifically regulated by 1, 2, 6, and 12 h of heat treatment, respectively (Figure 6, Table S2). Expressions of the remaining 386 DEGs were fluctuant with the time of treatments increase (other cluster), e.g., LOC_Os04g59440 was induced by WD_1 and WD_6, but not by WD_2 and WD_12.

### 2.6. Genes Involved in Multiple Biological Processes Are Responsive to Heat Stress

**Figure 7:**
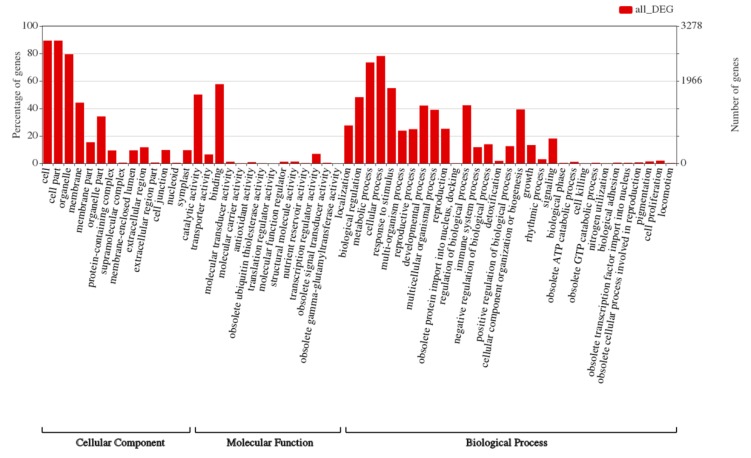
Enrichment of GO classification of 3559 differentially expressed genes.

To investigate which categories of genes may be involved in the thermotolerance of SDWG005, all detected DEGs were subjected to functional annotation and GO enrichment analysis. The results showed that the two most dominant GO terms in the molecular function category—catalytic activity and binding, and multiple biological processes, including metabolic process, cellular process, response to stimulus and biological regulation categories were significantly regulated by heat treatment (Figure 7). 

#### 2.6.1. Transcription Factors

Among the annotated DEGs, nine genes annotated as heat shock factors were upregulated by heat treatment, especially by the short-term treatment (WD_1). In addition to HSFs, a multiprotein bridging factor (LOC_Os06g39240) was significantly induced by all heat treatments. Moreover, more than 270 transcription factors responded to heat stress, predominantly 109 zinc finger-containing, 42 MYB, 41 F-box, 37 AP2/ERF superfamily, 22 bZIP, 14 helix-loop-helix DNA binding domain-containing, and 12 WRKY genes. However, the expression of most of these genes was repressed by heat stress.

#### 2.6.2. Nucleic Acid Synthesis and Modification

About 229 genes involved in nucleic acid synthesis and modification were identified as DEGs in this study. Of these genes, 139 were annotated as putative proteins containing pentatricopeptide repeats, which were mainly upregulated in the intermediate (WD_6) and long-term heat treatments (WD_12) (Table S2). It is notable that 73 genes were also induced specifically by these two heat treatments that participated in RNA biosynthesis and metabolism, including reverse transcriptase, RNA helicase, RNA polymerase, RNA recognition motif, RNA methylase, and tRNA synthetase genes. 17 DEGs encoding DNA polymerases, DNA mismatch repair proteins, DNA ligases, DNA-directed RNA polymerases, and exonucleases were also detected. In addition to the above genes, the expression of other genes essential for DNA and RNA synthesis and repair, such as ABC transporter and ATPase genes, were altered by the heat treatments in our study.

#### 2.6.3. Protein Synthesis and Posttranslational Modification

The expression of about 220 genes that participate in translation, including ribosome biogenesis, translation initiation and elongation, was altered by heat stress. For instance, 35 genes related to ribosomal proteins and ribosome biogenesis were upregulated specifically by WD_6 and WD_12, but not by the short-term heat treatments, WD_1 and WD_2. Furthermore, 28 ubiquitin metabolism-related genes were inducible under heat treatment, such as U-box domain-containing protein, ubiquitin carboxyl-terminal hydrolase, ubiquitin-conjugating enzyme, and ubiquitin-protein ligase genes. Amino acid permeases, amino acid transporters, and proton-dependent oligopeptide transporter (POT) family members involved in the transport of amino acids into cells were also identified as DEGs. It is telling that not only was protein synthesis affected by heat stress, but regulation of protein degradation also occurred under heat stress.

Among the DEGs observed in this study, 144 and 44 were annotated as protein kinases and phosphatases, respectively, according to the Nr database. Interestingly, 113 of the 144 protein kinases were tyrosine kinases. Moreover, these heat-responsive phosphatases mainly included protein phosphatase 2C proteins. In addition to phosphorylation, some genes participating in acylation and methylation were also heat-responsive in SDWG005 anthers, including histone deacetylase, acyltransferase, acyl carrier protein and methyltransferase genes.

As expected, the expression of 23 heat shock protein genes was similar to that of the HSFs, being upregulated by at least one of the heat treatments; more than half were small heat shock proteins, which were distinctly induced by 1 h of heat stress. In addition to the heat shock proteins, other chaperone proteins were heat-responsive in our study, including *ClpB1*, *DnaJ* domain-containing genes, and peptidyl-prolyl cis-trans isomerase.

#### 2.6.4. Physiological Processes Involved in the Heat Stress Response in Rice Anthers

Heat stress damages cell membranes in plants, and causes ion leakage from cells. Fatty acids are important components of the cell membrane. Hence, the relative electrical conductivity and concentration of fatty acids are commonly used as indicators of thermotolerance. In this study, we noted that six genes involved in fatty acid metabolism, including fatty acid desaturase and hydroxylase genes, were responsive to heat stress. In addition, 39 genes associated with ion transfer were regulated, such as heavy-metal-associated domain-containing, sodium/hydrogen exchanger, iron transporter, potassium channel, K^+^ potassium transporter, magnesium transporter, and sodium/hydrogen exchanger genes.

Phytohormones play a central role not only in plant development but also in biotic and abiotic stress responses. 26 hormone synthesis and signal transduction-related genes were identified as heat stress responsive, encoding proteins responsible for auxin synthesis signal transduction (inositol-3-phosphate synthase, auxin response factor, auxin-responsive protein, AUX/IAA family gene), gibberellin-regulated proteins, and proteins related to ethylene insensitivity (Table S2).

Our previous physiological study suggested that the excellent tolerance of SDWG005 to heat stress at the seedling stage be attributed to its strong ability to degrade reactive oxygen species (ROS) under heat treatment. Consistent with this notion, about 126 genes responsible for the clearance of ROS or involved in antioxidant defenses showed altered expression levels under heat stress, such as glutathione S-transferase, oxidoreductases, P450, peroxidase, thioredoxin, cysteine protease, and dehydrogenase genes.

As one of the most heat-sensitive physiological processes in the plant kingdom, the expression of photosynthesis-related genes was inhibited by heat stress in this study, especially by the prolonged heat treatments. For example, 19 chlorophyll A-B-binding proteins were induced by 1 h of heat stress but repressed by 6 and 12 h of heat stress. We observed a similar expression pattern for other 22 genes involved in photosynthesis, such as photosynthetic reaction center protein, oxygen-evolving enhancer protein, and photosystem I and II reaction center subunit genes.

Carbohydrates play important roles in anther and pollen development during heat stress. In this study, 67 glycosyl hydrolase genes, 24 UDP-glucosyl transferase genes and 18 glycosyltransferase genes were regulated by heat stress. Additionally, our study showed that 42 genes participating in sugar or glucose metabolism and other types of carbohydrate metabolism were regulated by heat stress, such as genes annotated as sugar transporters, trehalose-phosphatases, glucose/sorbosone dehydrogenases, alpha/beta hydrolases, and galactosyl transferases. It is worth mentioning that most of these genes were down-regulated.

### 2.7. Characterization of Anther Specific Gene OsACT

**Figure 8:**
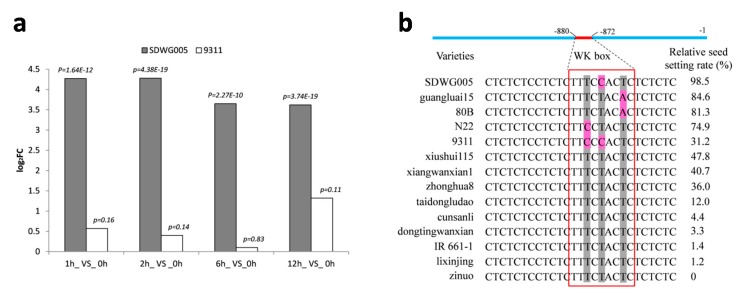
Expression and polymorphic analysis of *OsACT*. (**a**) Expression levels of *OsACT* in anthers of SDWG005 and 9311 under heat treatments by qRT-PCR. (**b**) Polymorphic analysis of WK-box in SDWG005, 9311, N22, and rice core collections with different thermotolerance. Pink indicates minor allele, gray indicates major allele; Relative seed setting rate data was from Zha et al. [20].

To further find some candidate genes that may be involved in rice thermotolerance, real time PCR was also conducted on expression of some DEGs in anthers of 9311 with the same heat treatments as SDWG005. It was revealed that *OsACT* (LOC_Os03g47860) showed quite different expression pattern between SDWG005 and 9311 under heat treatments (Figure 8a). Then, further analysis was conducted to understand the role of this gene in heat stress response. In silico study showed that this *OsACT* gene expressed specifically in anther and 5 DAPs’ seeds (http://rice.plantbiology.msu.edu), and our analysis suggested that there was no detectable expression of *OsACT* in leaves of SDWG005 and 9311 at seedling stage either in control or high temperature condition (Data not shown). Coding region of *OsACT* was 1389 bp in length without intron, and the putative protein sequence contained 462 amino acids. The encoded sequence contained the HXXXD domain and DFGGG domain for BAHD family at C-terminal and N-terminal, respectively. 

*OsACT* was further cloned from SDWG005 and 9311, and it was found that there were one and three SNPs in 5′ up-stream and coding region of *OsACT* between them, respectively. What is more, all the three SNPs in coding region lead to change of amino acid, but neither in HXXXD domain nor in DFGGG domain. However, the SNP (T/C) in 5′ up-stream region (−878 bp) located in the cis-element called WK-box (TTTTCCAC) [21]. Then, *OsACT* was cloned from 12 other lines with different thermotolerance ability, including N22 and 11 lines from rice core collections. As shown in Figure 8b, totally five haplotypes were detected for WK-box, including TCCACT, TCTACA, and CCTACT for thermotolerant lines, while CCCACT for 9311 and TCTACT for the other nine thermo-sensitive lines. 

## 3. Discussion

Extremely high temperatures are challenging for rice production, as heat stress usually reduces yields, along with the increasing global mean temperature and shortage of varieties with thermotolerance. For example, rice variety 9311 with its excellent agronomic trait performance has been widely planted in China. However, this variety set fewer seeds when subjected to heat stress at anthesis in this study, with a seed setting rate of only 31.2% [20]. Thermotolerant varieties are becoming a necessity for rice production.

Rice germplasm SDWG005, belonging to *Oryza sativa* L. spp. *xian*, is more tolerant to high temperature at the flowering stage than the well-known thermotolerant germplasm N22. In addition, SDWG005 was more thermotolerant at the seedling stage than 9311, as indicated by physiological and growth factors related to thermotolerance, such as photosynthesis and shoot and root fresh and dry weights. Therefore, SDWG005 has promise for breeding thermotolerant rice varieties, and N22 has been successfully used as a source of thermotolerance and drought tolerance in rice breeding [22,23].

It has been well documented that anthesis is the most temperature-sensitive stage in rice [10]. Many physiological processes during this stage are negatively influenced by heat stress, such as anther dehiscence, pollination, pollen germination on the stigma, and pollen tube growth to reach the ovule [24], resulting in reduced fertility. Thus, understanding the thermotolerance of anthers at anthesis is essential to elucidate thermotolerance in rice, which is directly associated with production under high temperature. Our morphological analysis showed that the anthers of SDWG005 and 9311 differ in their response to heat stress. Heat stress not only led to severe wilting of the anther sac wall in 9311 but also caused anther dehiscence without mature pollen release (Figure 2). However, SDWG005 anthers only slightly differed in the control and heat treatments (Figure 2). This finding suggests that heat stress damages rice anthers, particularly in the temperature-sensitive genotype 9311, because pollen development is the most sensitive process to heat stress. In rice, spikelet sterility occurs when temperatures exceed 35 °C for just 1 h [15]. Therefore, differences in the structure and response of SDWG005 and 9311 anthers under heat stress may explain their differences in the timing of seed set.

Transcriptional profiling analysis of stressed and unstressed plants would help to identify genes involved in acclimation and protection against heat stress. Since the relative seed setting rates of SDWG005 and 9311 differed, theoretical transcriptome analyses need to be performed for rice anthers. Although the current study focused on the dynamic gene expression profiles of anthers of the thermotolerant SDWG005 cultivar under heat treatment, we also analyzed the expression of some of these DEGs in 9311 under the same heat treatments using qRT-PCR. We found that these genes showed different expression patterns in SDWG005 and 9311 (Figure S3). This suggests that genotypes with different thermotolerances may respond differently at the molecular level. It was reported previously that induction levels of some heat-responsive genes in anthers correlated well with heat tolerance in rice [16]. Since both of the two studies focused on the genotypes with different genetic background, it was essential to conduct analysis on near-isogenic lines with different heat tolerances to illustrate the key factors responsible for thermotolerance of SDWG005 in the future.

In this study, 3559 genes were modulated at the transcription level under high-temperature stress; these genes were involved in various processes, including transcription regulation, nucleic acid synthesis and metabolism, protein synthesis and modification, hormone signal transduction, reactive oxygen species (ROS) elimination, and photosynthesis. All of these enriched functional categories have been associated with heat responses or thermotolerance in many species, including rice, wheat, maize, tomato, barley, and brassica [11,12,25,26,27,28], suggesting a relatively conserved mechanism in response to this kind of stress. For example, heat shock proteins (HSPs) are the most commonly detected molecules and act as an intermediate in protein folding or determination of the protein conformation during stress [29,30]. Moreover, the expression of HSPs and various other heat-responsive genes is controlled by heat shock transcription factors (HSFs) [31]. In the current study, most of the genes annotated as HSP (e.g., LOC_Os04g01740, LOC_Os03g14180, LOC_Os01g42190 and LOC_Os05g35400) and heat shock factors (e.g., LOC_Os05g49310, LOC_Os10g03730, LOC_Os02g34260) were also significantly induced by heat stress.

Since other studies have focused on heat-responsive gene profiles in rice reproductive tissues, including anthers [17] and pollinated pistil [16], common or specific responsive genes were identified by comparing our results to those of previous studies. We detected 48 heat-responsive genes in rice anthers, as done by Li et al. [17] and in our study, and 168 common DEGs in rice anthers (this study) and pollinated pistils under heat treatment [16] (Table S3). However, only 20 DEGs overlapped in the three studies mentioned above, 13 of which were HSPs, as expected (Table 1). These 20 genes are probably a core gene set that responds to heat stress in the floral organs of rice. Moreover, the few overlapping DEGs suggested that the response of rice to heat treatments varies greatly and could be dependent on the stress duration, tissue, or genotype.

Recent years have witnessed a breakthrough in the analysis of the molecular mechanisms of thermotolerance in plants. Increases or decreases in some genes could enhance the thermotolerance of rice or other plant species, which could be used to improve thermotolerance using modern breeding methods (reviewed by Grover et al. [32]). As mentioned above, LOC_Os03g47860 (*OsACT*) was significantly upregulated in thermotolerant SDWG005 at all time points examined under high-temperature stress but not in 9311 (Figure 8a). The *OsACT* gene may specifically express at the reproductive stage according to in silico data and real-time PCR data. Actually, in order to illustrate whether these DEGs were anther specific or not, we also analyzed expressions of the ten genes mentioned above in heat treated leaves in SDWG005 and 9311 at seedling stage. It was very interesting that only four of the ten genes were identified to be responsive to heat stress at seedling stage both in SDWG005 and 9311, however, the other six genes could not be detected either under the control or high temperature condition in both SDWG005 and 9311 at the seedling stage. It was worth mentioned that three of the four genes were heat shock proteins genes, which indicated that this kind of genes may be important to heat response and tolerance in the whole life of rice. While the other six genes could not be detected in leaves at seedling stage, suggesting that there may be unique mechanisms related to heat tolerance at certain development stages.

On the other hand, agmatine coumarin acyltransferase (ACT) is a member of the BAHD acyltransferase family that is unique to plants and participates in the acylation of phenolamides [33]. Phenolamines are not only involved in pollen development but also in resistance to abiotic stresses such as extreme temperature, drought, high salinity, and UV [34,35,36]. Phenolamine, as a substrate of peroxidase, also participates in the scavenging of hydrogen peroxide and strengthening of the extraplastid cell wall, so it functions as an antioxidant and free radical scavenger, which can improve the ability of plants to resist abiotic stress [37]. The most important is that spermidine, a precursor in phenolamine synthesis, can enhance thermotolerance in rice seeds by modulating endogenous starch and polyamine metabolism [38]. Therefore, to characterize the possible role of the *OsACT* in thermotolerance of rice anther at anthesis, this gene was cloned and sequenced from SDWG005, 9311 and N22. Sequences of *OsACT* were compared between SDWG005, 9311, N22, and other 11 rice core collections with different thermotolerance. The results showed that there were five haplotypes in WK-box in promoter region of *OsACT* (Figure 8b), which was located in the −872 bp to −880 bp upstream region. It was interesting that the core sequence in 9 out of the 10 thermo-sensitive lines was TCTACT, except for 9311, nevertheless, a considerable variation in WK-box was observed in these thermotolerant lines (Figure 8b). WK-box has been reported to be WRKY12 binding site in tobacco [21]. Variation of WK-box in these accessions indicated that *OsACT* may play an important role in anther thermotolerance during rice anthesis. Functional analysis of *OsACT* is still essential to investigate its importance in thermotolerance of rice.

Overall, a dynamic heat-responsive transcriptome analysis of the anthers of the thermotolerant rice cultivar SDWG005 provided the basis for illustrating the molecular mechanism of its thermotolerance. On the other hand, the DEGs identified in this study pave the way for further research regarding the use of heat-tolerant genes in rice breeding, aimed at improving the performance of rice varieties under challenging environments.

## 4. Materials and Methods 

### 4.1. Plant Material and Experimental Treatments

Two rice (*Oryza sativa* L. spp. *xian*) genotypes, SDWG005 and 9311, were used in this study. SDWG005 is an African landrace and 9311 is a widely used restorer line in China. The seeds were soaked in water at 28 °C for 24 h and subsequently incubated at 28 °C for 48 h for germination. The germinated seeds were transferred to 96-well plates filled with vermiculite with one seedling per well. The seedlings were irrigated with distilled water until the two-leaf stage and Yoshida solution thereafter [39] (pp. 53–57). The 30-day-old seedlings were transplanted into plastic barrels (30 cm diameter) filled with paddy soil. Three plants were planted in each barrel, with five tillers retained for each plant. The seedlings and plants were grown in a growth chamber with a relative humidity of 75% and a natural photoperiod.

There were two temperature treatments: (1) control, 28 °C during the day (6:00 am to 6:00 pm) and 22 °C at night (6:00 pm to 6:00 am); and (2) heat stress (HS), 38 °C during the day (6:00 am to 6:00 pm) and 28 °C at night (6:00 pm to 6:00 am).

### 4.2. Microscopic Observations of Rice Anthers

Florets of each genotype in the control and heat stress treatment were subjected to microscopic observation. Anthers which ascending to the top of glume from the middle part of the first branch in every genotype under different treatments were carefully collected using tweezers. Some of these anthers were morphologically observed under a VHX-2000 digital microscope (Keyence, Osaka, Japan), with the remainder fixed in a 50% FAA solution (40% formalin:glacial acetic acid:50% ethanol = 1:1:18) and then dehydrated with 100% ethanol, followed by washing with xylene. The anthers were embedded in paraffin before being sectioned (thickness, 10 microns) and stained with safranine O-Fast Green to produce permanent slices to examine the microstructure of anther sacs under a NIKON ECLIPSE E100 positive feedback microscope (Nikon, Tokyo, Japan).

### 4.3. Seed Setting Rate

After maturation, the marked spikes from ten plants were harvested separately for each genotype within each treatment. The number of empty and filled grains was recorded, with the seed set rate represented as the percentage of filled grains in all grains. The effect of heat stress on seed set was indicated by the relative seed setting rate, being seed setting rate at high temperature/seed setting rate at normal temperature × 100%.

### 4.4. Sample Collection for RNA-Seq and Real-Time PCR

A total of 75 plants per genotype were used for sample collection. When three or more panicles extended from flag leaves by approximately 2 cm, the panicles were labeled, and the plants transferred to the growth chamber for the HS treatment. The labeled spikes were harvested before HS treatment (0 h) and 1, 2, 6, and 12 h after the initiation of the HS treatment, respectively, and placed on ice before anther extraction. The treatments were designated WD_0 and 9311_0, WD_1 and 9311_1, WD_2 and 9311_2, WD_6 and 9311_6, and WD_12 and 9311_12 for SDWG005 and 9311, respectively. One hour and 2 h were considered as short-term, 6 and 12 h were considered as intermediate and long-term heat treatments, respectively. For each treatment, 15 independent plants were sampled, and mature anthers which ascending to the top of glume from five independent plants pooled as one biological replicate, such that there were three biological replicates per treatment. The labeled panicles from each time point in each treatment were collected on ice, with the mature anthers from the panicles isolated, immediately suspended in liquid nitrogen, and stored at −80 °C until RNA extraction.

### 4.5. Total RNA Extraction and RNA Sequencing

Total RNA was extracted from each sample using an RNAprep Pure Plant Kit (Tiangen Biotech, China) following the manufacturer’s instructions. The quality and quantity of RNA samples were assessed by gel electrophoresis and a Nanodrop (Thermal Fisher, Waltham, MA, USA). The resultant 15 RNA samples for SDWG005 were used for RNA-seq analysis. Upon treatment with DNase I, 1 µg of RNA from each sample was used for sequencing library preparation with a NEB Next Ultra TM RNA Library Prep Kit for Illumina (NEB, Ipswich, MA, USA). Library preparations were sequenced from paired ends to obtain 150 bp reads (PE150) on an Illumina platform.

### 4.6. Quality Control and Comparative Analysis of RNA-seq Data

Raw data were processed through in-house Perl scripts to remove low-quality reads (more than 20% of bases presenting a Q value ≤ 20 or ambiguous sequence content (“N”) exceeding 5%). Clean reads were then obtained by removing reads containing adapters and poly-N sequences. Clean reads were mapped to the rice reference genome sequence (MSU7.0, http://rice.plantbiology.msu.edu/) with Hisat2 tools [40]. Only reads with a perfect match or one mismatch were further analyzed. 

### 4.7. Differential Expression Analysis

Gene expression levels were quantified as fragments per kilobase of transcript per million fragments mapped (FPKM) values. Pearson’s correlation coefficient [41] was used to calculate the correlation coefficients of FPKM of all transcripts among three replications for each sample. Differential expression analysis of the two conditions was performed using DEseq2 [42]. The resulting *p* values were adjusted using the Benjamini and Hochberg approach [43] to control the false discovery rate. Genes with an adjusted *p* < 0.05 and |log_2_FC| > 1 identified by DEseq2 were assigned as differentially expressed. Hierarchical clustering analysis of DEGs was conducted using cluster *R* package based on lgFPKM of them among three replications for each sample (https://cran.r-project.org/web/packages/cluster). Heatmap was plotted by pheatmap *R* package (https://cran.r-project.org/web/packages/pheatmap), Venn was plotted by Venndiagram [44] *R* package. Expression pattern of genes in different clusters were plotted using our in house scripts based on *R* language (File S1).

### 4.8. Quantitative Real-Time PCR

Quantitative real-time PCR (qRT-PCR) was applied to quantify the expression levels of 10 selected DEGs in SDWG005 and 9311. RNA for all the samples was reverse-transcribed using a RevertAid First Strand cDNA Synthesis Kit (Thermo Scientific) following the manufacturer’s protocol. qRT-PCR was performed using ABI StepOnePlus™ Real-Time System. Relative gene expression levels were calculated using the 2^−ΔΔ*C*t^ method, as described by Min et al. [45]. The expression of each replicate was normalized according to the reference gene *OsActin* (LOC_Os03g50885). For randomly selected genes, specific primers (listed in Table S4) were designed and tested by qRT-PCR. The mean of three replicates represents the relative expression level.

### 4.9. Gene Functional Annotation and Enrichment Analysis

Gene functions were annotated based on the following databases: Nr (non-redundant protein sequence database), Pfam (database of homologous protein family), KOG/COG (Clusters of Orthologous Groups of proteins), Swiss-Prot (manually annotated, non-redundant protein sequence database), KO (KEGG Ortholog) and GO (Gene Ontology database). GO enrichment analysis of the DEGs was implemented with clusterProfiler *R* packages [46]. We defined significant enrichment based on an FDR < 0.05.

### 4.10. Cloning and Sequence Analysis of OsACT Gene in Rice 

Coding region as well as upstream region of *OsACT* was extracted from Rice Genome Annotation Project (http://rice.plantbiology.msu.edu/) using LOC_Os03g47860 as the query. Six overlapped primer pairs (Table S4) were designed and used to amplify *OsACT* from genomic DNA of SDWG005, 9311 and N22. DNA was extracted using Plant DNA extraction kit according to the manufacture’s instruction (Tiangen, Beijing, China). Specific PCR product with expected size was subjected to Sanger sequencing (Tianyi, Wuhan, China). Sequences of *OsACT* in other 11 rice core collections were downloaded from http://www.rmbreeding.cn. Alignment of gene sequence was conducted using DNAMAN software (version 8.0, Lynnon Biosoft, San Ramon, CA, USA).

## Figures and Tables

**Table 1:** Common DEGs compared with the transcriptional profiles of the pollinated pistil [16] and anther [17].

Gene ID	WD_1		WD_2		WD_6		WD_12		Functional Annotation
	log_2_FC	*p* Value	log_2_FC	*p* Value	log_2_FC	*p* Value	log_2_FC	*p* Value	
LOC_Os11g13980	7.07	8.01 × 10^−5^	7.39	4.48 × 10^−18^	8.09	2.01 × 10^−31^	8.05	1.87 × 10^−29^	hsp20
LOC_Os03g14180	6.86	2.02 × 10^−17^	6.83	1.65 × 10^−134^	6.77	2.44 × 10^−99^	4.69	9.23 × 10^−32^	hsp20
LOC_Os04g01740	4.71	2.27 × 10^−28^	3.60	1.00 × 10^−38^	3.77	4.50 × 10^−43^	1.97	9.91 × 10^−9^	hsp90
LOC_Os04g36750	3.57	3.35 × 10^−22^	2.60	3.67 × 10^−18^	1.64	2.63 × 10^−5^	2.13	6.51 × 10^−5^	hsp20
LOC_Os02g52150	3.40	5.69 × 10^−32^	3.00	4.65 × 10^−40^	2.56	3.06 × 10^−24^	3.08	2.04 × 10^−5^	hsp20
LOC_Os04g45480	3.08	9.62 × 10^−21^	2.58	5.52 × 10^−54^	2.10	5.23 × 10^−20^	1.42	5.63 × 10^−6^	uncharacterized protein
LOC_Os03g16920	2.05	5.14 × 10^−7^	1.93	1.36 × 10^−18^	2.35	2.28 × 10^−20^	2.63	1.54 × 10^−12^	hsp70
LOC_Os04g28420	2.41	1.36 × 10^−41^	1.63	5.46 × 10^−21^	1.22	4.19 × 10^−8^	–	–	70 kDa peptidyl-prolyl isomerase
LOC_Os02g15930	1.94	1.90 × 10^−15^	1.07	2.10 × 10^−8^	1.42	1.23 × 10^−16^	–	–	uncharacterized protein
LOC_Os01g08860	3.05	1.20 × 10^−28^	1.88	5.74 × 10^−24^	–	–	−1.71	2.36 × 10^−9^	hsp20
LOC_Os11g05170	2.76	1.00 × 10^−11^	–	–	−2.15	5.63 × 10^−5^	−2.14	1.20 × 10^−3^	uncharacterized protein
LOC_Os11g32890	2.56	6.08 × 10^−20^	–	–	−1.68	7.10 × 10^−7^	−1.87	1.65 × 10^−7^	uncharacterized protein
LOC_Os02g54140	1.95	2.28 × 10^−7^	–	–	−1.80	8.55 × 10^−6^	−3.68	1.23 × 10^−17^	hsp20
LOC_Os01g55270	1.37	3.70 × 10^−12^	–	–	−1.31	1.22 × 10^−14^	−1.26	1.24 × 10^−13^	calcyclin-binding protein
LOC_Os01g04370	2.85	2.73 × 10^−17^	1.98	7.64 × 10^−19^	–	–	–	–	hsp20
LOC_Os03g16020	2.09	2.88 × 10^−7^	1.02	3.61 × 10^−5^	–	–	–	–	hsp20
LOC_Os05g46480	–	–	–	–	−2.58	1.42 × 10^−16^	−3.56	1.35 × 10^−27^	late embryogenesis abundant protein
LOC_Os03g16030	3.98	1.97 × 10^−3^	–	–	–	–	–	–	hsp20
LOC_Os03g16040	2.20	5.80 × 10^−12^	–	–	–	–	–	–	hsp20
LOC_Os01g18080	–	–	−1.11	1.33×10^−5^	–	–	–	–	uncharacterized protein

## Supplementary Materials

Supplementary materials can be found at https://www.mdpi.com/1422-0067/21/3/1155/s1. Figure S1. Cluster analysis of all samples based on lgFPKM of 3559 DEGs; Figure S2. Heatmap clustering of the global patterns of DEGs expressed at one or more time points in the heat treatment. The color scale represents the lgFPKM (Mean values of FKPM of three replicates); Figure S3. Expression levels of ten randomly selected DEGs by RNA-seq and qRT-PCR. RNA-seq: expression change of these genes in anthers of SDWG005 under heat treatments as compared to control by RNA-seq. WD-qPCR: expression change of these genes in anthers of SDWG005 under heat treatments as compared to control by real-time PCR. 9311-qPCR: expression change of these genes in anthers of 9311 under heat treatments as compared to control by real-time PCR; Table S1. Correlation of all the samples based on FKAM of all transcripts; Table S2. Differentially expressed genes (DEGs) at a minimum of time points during the heat treatment; Table S3. Differentially expressed genes (DEGs) compared with the transcriptional profiles of the pistils and anthers. Table S4. Primers for qRT-PCR and cloning of *OsACT*; File S1. *R* script for expression pattern of genes in different clusters.
