# Supplementary figures and images for: Dynamic Transcriptome Analysis of Anther Response to Heat Stress during Anthesis in Thermotolerant Rice (Oryza sativa L.)
Source: Int J Mol Sci. 2020 Feb 10;21(3):1155. doi: 10.3390/ijms21031155 (PMC7037497; doi:10.3390/ijms21031155)

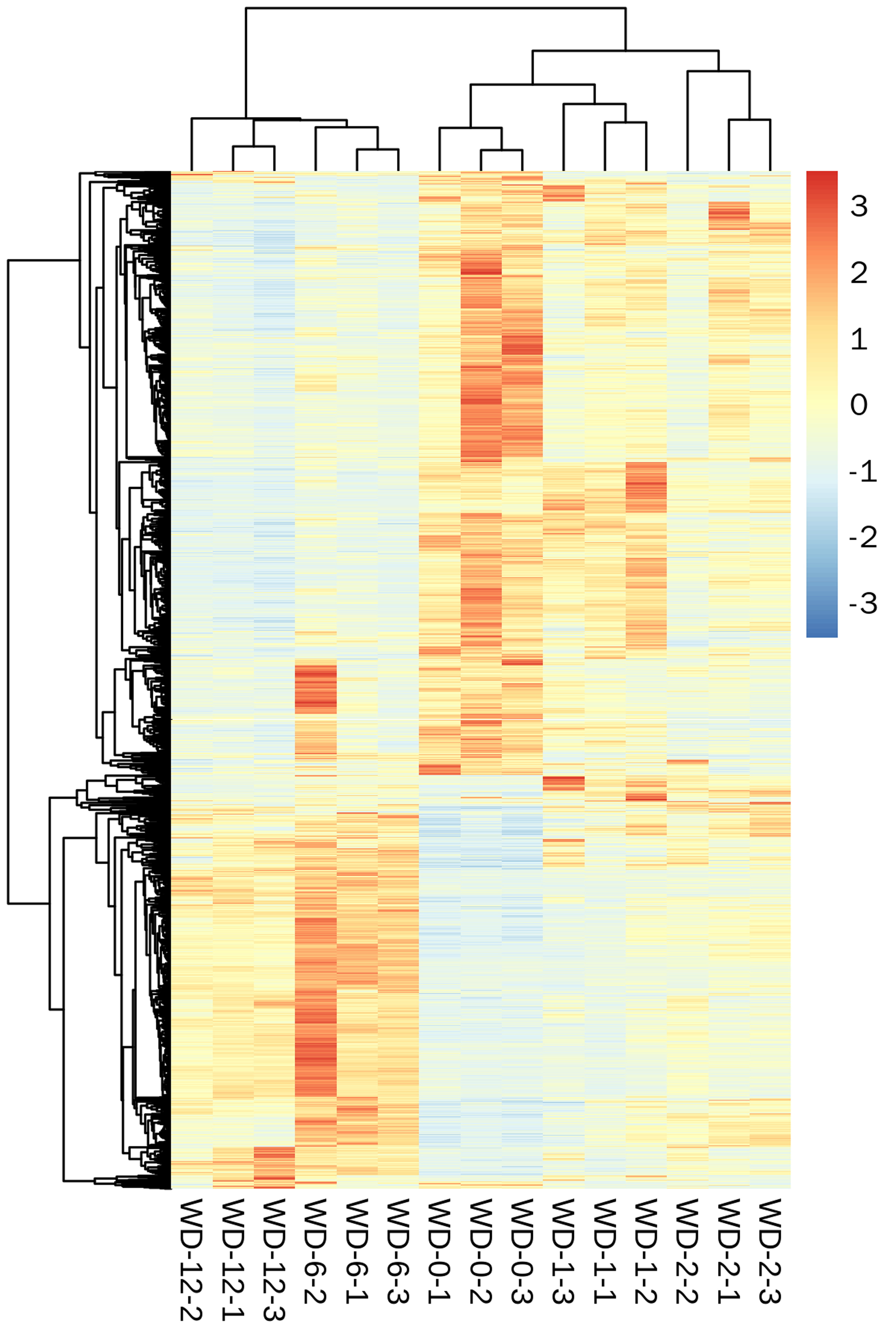

Supplement: Supplementary file 1 [file ijms-21-01155-s001.zip › supplementary file/Figure S1.tif]

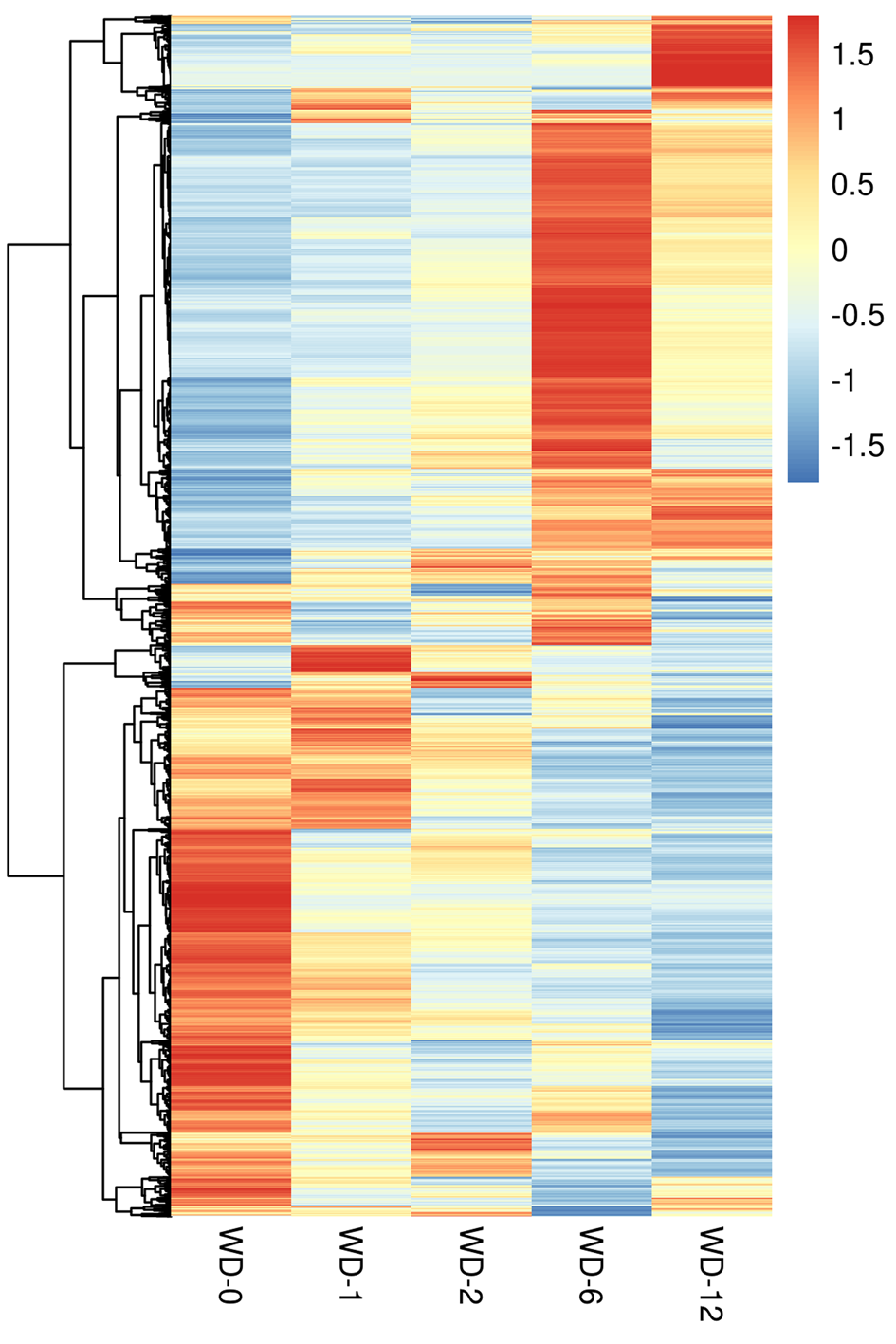

Supplement: Supplementary file 1 [file ijms-21-01155-s001.zip › supplementary file/Figure S2.tif]

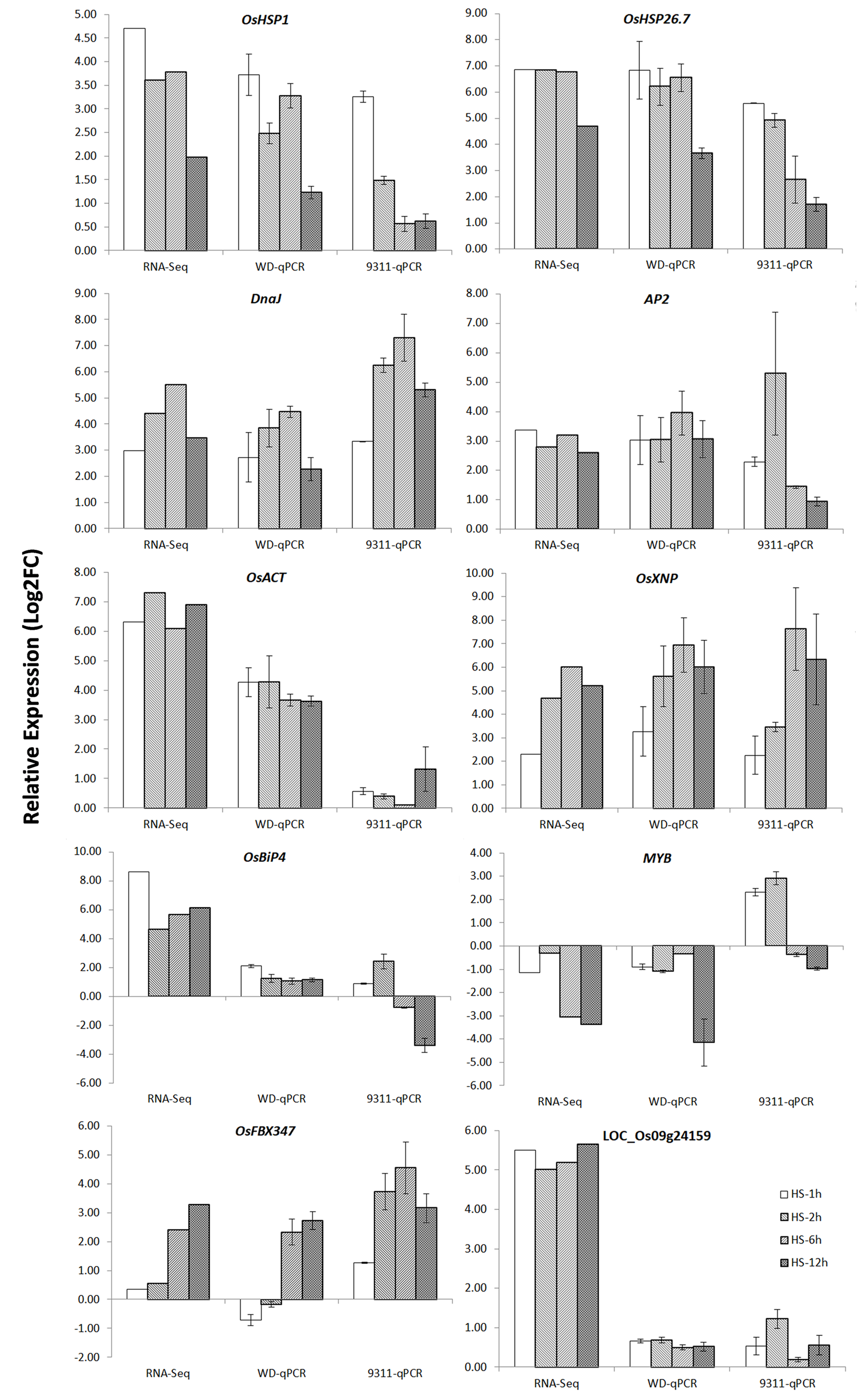

Supplement: Supplementary file 1 [file ijms-21-01155-s001.zip › supplementary file/Figure S3.tif]
